# Entering the new normal - psychosocial work environment and health during the transition from full remote to a hybrid work arrangement in a higher education context

**DOI:** 10.1371/journal.pone.0353162

**Published:** 2026-07-31

**Authors:** Jeffrey Casely-Hayford, Helena Tinnerholm Ljungberg, Emmanuel Aboagye, Christina Björklund, Lydia Kwak, Susanna Toivanen, Gunnar Bergström, Irene Jensen

**Affiliations:** 1 Unit of Intervention and Implementation Research for Worker Health, Institute of Environmental Medicine, Karolinska Institutet, Stockholm, Sweden; 2 Department of Psychology, Norwegian University of Science and Technology, Trondheim, Norway; 3 Department of Social Sciences and Humanities, Faculty of Philosophy, Mälardalen University, Västerås, Sweden; 4 Department of Occupational Health, Psychology and Sports Sciences, Faculty of Health and Occupational Studies, University of Gävle, Gävle, Sweden; Instituto Federal Goiano, BRAZIL

## Abstract

This longitudinal study explored the perceptions of psychosocial work factors, work-related health, and job satisfaction of 745 professional service staff, working in a higher education setting in Sweden, during the transition from full remote work to a hybrid work arrangement over 12-months. The study also explored whether these perceptions differed due to gender and household composition. The questionnaire data were analyzed using linear mixed-effects models with estimated marginal means and Bonferroni-adjusted pairwise comparisons. The results showed that while overall perceptions of psychosocial work factors showed modest changes during the transition, except for social support from colleagues, which significantly increased over time. Professional service staff reported higher levels of stress and exhaustion during the transition, while job satisfaction remained unchanged. The results also highlighted significant gender differences during the transition, as women reported less favourable perceptions of psychosocial work factors and higher levels of stress, while men’s perceptions remained largely stable across these outcomes. Significant overall differences in household composition were also observed, as individuals living in single-person households and in single-parent households reported less favourable perceptions of psychosocial work factors and their work-related health compared to cohabitants and cohabitants with children, though all household groups followed similar trajectories during the transition. The findings suggest that higher education institutions should implement action plans for gender equality and family-supportive practices in hybrid work arrangements to ensure that the benefits of hybrid work are accessible to all employees.

## 1. Introduction

The growing interest in flexible work arrangements stems from the significant shift to hybrid work models that accelerated during the COVID-19 pandemic, leading to long-term changes in workplace dynamics. A challenge in the literature is navigating the conceptual ambiguity between concepts describing various flexible work arrangements such as remote work, telework and hybrid work [1]. However, these concepts can be differentiated by their relation to a central workplace and by where the work is performed. Remote work refers to dependent employees performing tasks using various technologies outside a central workplace, which offers sub-work arrangements reflecting varying degrees of flexibility and work organization [2]. Telework refers to a form of performing and organizing remote work away from a central workplace during normal working hours, with varying degrees of physical and temporal constraints, using information and communications technologies (ICT) [1]. Hybrid work is a more distinctive form of telework that blends traditional and non-traditional work practices, allowing for both in-office collaboration and remote autonomy [3,4]. It differs from standard telework by incorporating structured flexibility, enabling employees to alternate between physical and virtual workspaces based on predefined arrangements, and it entails a greater degree of temporal and spatial flexibility as it involves both synchronous and asynchronous work as well as a high degree of multi-locational work [1]. According to Lauring and Jonasson [5] hybrid work “*entails ongoing switches between interrelated traditional and non-traditional work modes in relation to modality, location, and temporality where an equal balance between working in traditional and non-traditional work modes represents the highest level of hybridity*”. Although the prevalence of telework increased during the outbreak of the COVID-19 pandemic, the prevalence of telework was comparatively high in the Nordic countries, such as Sweden, before the pandemic [6]. In 2019, around ~35% of employees in Sweden reported teleworking to some degree, with this figure increasing to ~45% during the pandemic [1]. In 2022, the prevalence of telework in Sweden remained high, and five of the twenty EU regions with the highest rates of telework were in Sweden [7].

Given its prevalence in Sweden and across the Nordic countries, understanding how telework influences employees is particularly relevant in sectors where it is most established. Telework is most common in office-based, knowledge-intensive work, particularly in public sector organizations, business services, higher education, and research, where operational flexibility supports remote work [8,9]. Jobs within these sectors are characterized by a high degree of autonomy, managerial trust, and workplace flexibility, which diminishes the spatial and temporal constraints of employees’ work tasks [10]. The widespread use of telework has led to an interest in how it influences employees’ perceptions of their psychosocial work environment and employees’ work-related health. The loosening of the temporal constraints of work has been shown to influence employees’ perception of their psychosocial work environment as it places more responsibility on the employee to regulate their working time [6]. Although this provides employees with a greater degree of autonomy and control, it also paves the way for boundaryless working hours and work intensification [11,12]. The literature has also shown how the spatial component of telework yields differential outcomes. Whereas the multi-locationality enabled by telework can facilitate favourable employee outcomes such as better work-life balance and higher levels of work engagement [13], it can also induce feelings of professional isolation by negatively impacting employees’ sense of connectedness to their colleagues and supervisors [14]. However, a recent systematic review by Vleeshouwers et al. [13] concluded that the relationship between telework and employees’ perception of their psychosocial work environment is far from clear and underexplored. As telework may influence employees’ ability to psychologically detach from work, it may also impact their work-related health [15]. However, the influence of teleworking on work-related health outcomes such as stress and exhaustion is still unclear due to the few studies exploring these health outcomes and the lack of longitudinal studies investigating the relationship [16,17]. The unclear relationship between teleworking, employees’ perceptions of their psychosocial work environment, and work-related health may stem from the conceptual ambiguity in distinguishing various forms of teleworking in the literature. A recent systematic review by Mele et al. [18] called for a sectoral perspective as public and private sector organizations diverge in key contextual ways, which are of relevance when exploring the work-related effects of teleworking. The review by Mele et al. [18] included 120 studies set in public sector organizations, such as public universities and governmental organizations, and examined the work-related effects of telework in public sector organizations. The findings showed that the outcomes most frequently investigated related to individual productivity and work-life balance, which further highlights the lack of studies examining the relationship between telework and employees’ perceptions of the psychosocial work environment. The literature has also directed attention towards gender differences in telework-related effects [19,20]. A systematic review by Crawford [21] found that female employees who teleworked reported higher levels of workload, workaholism, exhaustion, and stress compared to their male counterparts. A recent systematic review by Castro-Trancón et al. [22] examined the work-related effects of telework from a gendered perspective. Of the 35 included studies, 22 studies found that telework had a greater negative work-related impact on women, as women reported higher levels of workload, stress, depressive symptoms, as well as lower levels of job satisfaction. Previous studies have suggested that women are more negatively affected by telework as they find it more difficult to establish boundaries between their work and family life [23]. As such, Castro-Trancón et al. [22] suggested that the gender differences found in the telework literature may intersect with household composition and are best explored through the lens of work-life conflicts.

While the broader telework literature highlights these concerns around psychosocial work factors, work-related health and gender differences, comparatively less is known about these associations within specific organizational contexts. The higher education system provides an interesting setting to study the work-related effects of telework as it has undergone extensive changes, driven by increasing calls for efficiency and productivity, which have reshaped the organizational cultures found within higher education institutions [24,25]. This culture of performativity has contributed to a deterioration in the working conditions and psychosocial work environment found in higher education institutions [26]. Research on telework in higher education is limited. However, some studies suggest that teleworking can improve the psychosocial work environment and work-related health outcomes among staff (e.g., 24). However, studies have also found an association between telework and higher levels of stress among research staff [27,28]. Few studies have focused on the work-related effects of teleworking among professional service staff, who represent a large proportion of the employees at higher education institutions [29]. A study by Watermeyer et al. [30] examined the experiences of teleworking among 4731 professional service staff in the UK and found that, despite reporting an increase in their job demands, most staff did not experience a change in their collegial relationships or with their work-related health while working remotely. A study by Ghislieri et al. [31] comparatively examined the experiences of telework among 2365 research staff and 4086 professional service staff in Italy. In the study, professional service staff reported lower levels of workload, workaholism, and exhaustion compared to research staff. However, the findings highlighted gender differences in the work-related effects of telework among both research and professional service staff, as women in both sub-samples reported higher levels of negative work-related effects.

Despite the growing body of literature on telework, significant gaps remain regarding how the transition to hybrid work arrangements affects psychosocial work factors, work-related health, and job satisfaction among professional service staff. The aim of this paper is to explore the work-related associations of hybrid work among professional service staff in a higher education context. Specifically, this paper explored the overall perceptions of psychosocial work factors, work-related health, and job satisfaction among professional service staff working in a higher education setting in Sweden during the transition from full remote work to a hybrid work arrangement over 12-months. Furthermore, this paper also explored the influence of gender and household composition on these perceptions during the transition to a hybrid working arrangement. As such, this paper aims to address important empirical gaps in the literature by examining how perceptions of psychosocial work factors, work-related health, and job satisfaction change during the transition from full remote work to a hybrid work arrangement over 12-months using an understudied population – professional service staff. The specific research questions addressed in this paper are:

1. Are there any changes in the perceptions of psychosocial work factors, work-related health, and job satisfaction among professional service staff during the transition from full remote work to a hybrid work arrangement over 12-months?
2. Are there any gender differences in the perception of psychosocial work factors, work-related health, and job satisfaction among professional service staff during the transition from full remote work to a hybrid work arrangement over 12-months?
3. Are there any household composition differences in the perception of psychosocial work factors, work-related health, and job satisfaction among professional service staff during the transition from full remote work to a hybrid work arrangement over 12-months?

## 2. Method

### 2.1 Study design and data collection procedure

Discussions about transitioning to a hybrid work arrangement were initiated at the university in November 2020. Hybrid work referred to the possibility for all staff to work remotely up to 49% of their working hours, with the extent and nature of this subject to the approval by employees first-line manager. The hybrid work arrangement employed at the university, therefore, aligned with the abovementioned definition of hybrid work as a blend of traditional and non-traditional work practices characterized by a high degree of temporal and spatial flexibility [5]. The current study is a longitudinal study based on questionnaire data collected between August 2021 and April 2023. Ethical approval for the study was granted by the Swedish Ethical Review Authority (Dnr 2021−03637). Written consent was obtained from the participants in the questionnaire. Data was collected among professional services staff at a medical faculty in Sweden using a web-based questionnaire at ten timepoints. Data was collected using a condensed version of the questionnaire at seven of these ten timepoints. Table 1 presents an overview of the data collection in relation to the Swedish national recommendations during the COVID-19 pandemic. The current study is based on three timepoints that capture the transition from full remote work to a hybrid work arrangement: Baseline (August 2021; T0), 3-month follow-up (October 2021; T1), and 12-month follow-up (September 2022; T2). The data collected at T0 (baseline) occurred at a time when full remote work was recommended by the Swedish Government due to the COVID-19 pandemic. The data collected at T1 (3-months) occurred at a time when the national recommendations for full remote work had been lifted, and employees went back to work in a hybrid work arrangement. The data collected at T2 occurred 12 months after baseline and captures a period in which employees had been working in a hybrid work arrangement for 9 months – as the transition to a hybrid work arrangement started at T1.

**Table 1:** Overview of data collection procedure in relation to the work arrangements at the faculty and the national recommendations during the COVID-19 pandemic.

Timepoints in the current study	Timepoints in the overall data collection	Recommendations/change in recommendations
**T0**	**T1: August 2021**	Full remote work
	**T2: September 2021**	Full remote work Recommendations for full remote work were lifted on the 29/9/2021
**T1**	**T3: October 2021**	Hybrid work
	**T4: November 2021**	Hybrid work Recommendations for full remote work were reinstated on the 23/12/2021
	**T5: January 2022**	Full remote work Recommendations for full remote work were lifted on the 09/02/2021
	**T6: March 2022**	Hybrid work
	**T7: June 2022**	Hybrid work
**T2**	**T8: September 2022**	Hybrid work
	**T9: November 2022**	Hybrid work
	**T10: April 2023**	Hybrid work

### 2.2 Study sample

The current study uses a sub-sample consisting of 745 professional service staff, which does not include managers. Table 2 provides a descriptive overview of the study sample, which shows that most of the study sample were female (75%); cohabitants with children (49%); employed on permanent contracts (93%); and worked with a variety of professional services.

**Table 2:** Descriptive overview of study sample.

Variable	
**Gender, n (%)**	
Women	555 (75%)
Men	187 (25%)
**Age,** *m (sd)*	47.1 (10.2)
**Household composition, n (%)**	
Single-person household	115 (15%)
Single-parent household with children	62 (8%)
Cohabitant	206 (28%)
Cohabitant with children	362 (49%)
**Branch of professional services**	
Communication and public relations office	40 (5%)
Property and facilities office	11 (1%)
Education support office	135 (18%)
Library services	48 (6%)
Research support unit	60 (8%)
Legal office	22 (3%)
Finance office	94 (13%)
IT	94 (13%)
Human resource office	62 (8%)
Administrative support	148 (20%)
Other professional services	31 (4%)
**Employment terms**	
Permanent	694 (93%)
Fixed term	51 (7%)
**Years working at current workplace**	
Less than 5 years	368 (49%)
5 years or more	377 (51%)

### 2.3 Measures

#### 2.3.1 Psychosocial work factors and job satisfaction.

The Copenhagen Psychosocial Questionnaire III (COPSOQ III; [32,33]) is a validated risk assessment instrument that was used to measure four psychosocial work factors: quantitative demands (assessed using three questions, e.g., *Is your workload unevenly distributed so it piles up?*); Social support from colleagues (assessed using one question, *How often do you get help and support from your colleagues, if needed?*); Social support from supervisor (assessed using one question, *How often do you get help and support from your immediate superior, if needed?*) and Influence at work (assessed using four items, e.g., *Do you have a large degree of influence on the decisions concerning your work?*). Although the full social support from supervisor and social support from colleagues’ scales consist of three questions each, the present study only included the mandatory core item in each scale, which is required when using shortened versions of COPSOQ scales [33]. The COPSOQ III was also used to measure job satisfaction which was assessed using one question (*Regarding your work in general, how pleased are you with: your job as a whole, everything taken into consideration?*). All the COPSOQ scales were measured using a 5-point Likert scale and scaled to the interval 0, 25, 50, 75, 100 in accordance with the COPSOQ questionnaire [33]. The 0–100 scoring in the COPSOQ questionnaire is used to ease practical interpretation of the results to make group differences more intuitive and to enable benchmarking against reference values [32,33].

#### 2.3.2 Work-related health factors.

The COPSOQ III was also used to measure one work-related health factor: physical and mental exhaustion (hereafter referred to as exhaustion; assessed using three questions, (e.g., *How often have you been emotionally exhausted?*). The other work-related health factor stress was assessed using the Swedish version of a validated single-item question on a 5-point Likert scale (*Stress means a situation in which a person feels tense, restless, nervous or anxious or is unable to sleep at night because his/her mind is troubled all the time. Do you feel this kind of stress these days?*; [34]). The COPSOQ scale for exhaustion were measured using a 5-point Likert scale and scaled to the interval 0–100, in accordance with the COPSOQ questionnaire [33]. The single-item question assessing stress was also scaled to the interval 0–100 to ensure comparability across all outcomes. A Cronbach’s alpha test was conducted for the multi-item scales used in the study, showing a range from 0.76 to 0.89, indicating good reliability [35].

**Table 3:** Descriptive overview of the scales and measures.

	Baseline		3-months		12-months	
	Mean (sd)	α	Mean (sd)	α	Mean (sd)	α
***Psychosocial work factors***						
Quantitative Demands ^a^	40.35 (18.04)	.76	43.93 (18.81)	.78	–	–
Social support from supervisor ^b^	78.50 (24.68)	–	74.86 (27.30)	–	78.15 (24.61)	–
Social support from colleagues ^b^	81.09 (20.49)	–	78.28 (22.16)	–	82.90 (19.59)	–
Influence at work ^b^	50.36 (18.52)	.78	49.86 (18.71)	.77	51.44 (18.20)	.81
***Work-related health factors***						
Exhaustion ^a^	24.62 (20.11)	.87	28.93 (22.09)	.89	26.47 (20.02)	.82
Stress ^a^	34.19 (25.65)	–	40.66 (28.84)	–	41.14 (28.80)	–
***Job Satisfaction***						
Job Satisfaction ^b^	77.47 (18.08)	–	75.42 (19.14)	–	75.47 (18.20)	–

Note: ^a^ = higher scores indicate higher demands, exhaustion, and stress ^b^ = Higher scores indicate higher levels of support and influence at work.

#### 2.3.3 Individual factors.

Gender was measured as a polychotomous variable (male, female, other), and two people responded as “other”. However, as their inclusion could potentially breach the confidentiality and anonymity of their responses, by increasing the risk of re-identification, these individuals were excluded, and gender was therefore treated as a dichotomous variable. Household composition was assessed as a polychotomous variable with four groups: single-person household, single-parent household with children, cohabitant, and cohabitant with children.  Table 3 provides a descriptive overview of the scales used in the study.

### 2.4 Analytical approach

To examine how perceptions of psychosocial work factors, work-related health and job satisfaction developed during the transition from full remote to hybrid work, longitudinal data were analysed using linear mixed-effects models. This modelling approach was used because it enables the estimation of changes over time while accounting for the correlation between repeated measurements within individuals and allowing all available observations to contribute to the analyses under a missing at random (MAR) assumption. Time was included as a categorical fixed effect to capture potential non-linear changes across the three measurement points during the 12-month transition period. Gender and household composition were included as time-invariant fixed effects to explore overall group differences. Interaction terms between time × gender and time × household composition were specified to assess differences in the perception of psychosocial work factors, work-related health and job satisfaction among individuals during the transition. As such, the main effect of time captures overall changes across timepoints while the main effects of gender and household composition capture overall group differences averaged across timepoints, whereas the specified interaction effects capture whether the pattern of change over time differed between gender and household composition groups.

To account for the fact that repeated measurements closer in time are likely to be more similar, a first-order autoregressive covariance structure was specified. This covariance structure was used as it provided a reasonable and stable representation of within-person correlations over time. Models were estimated using restricted maximum likelihood with Satterthwaite-adjusted degrees of freedom. Estimated marginal means were used to facilitate interpretation of overall time trends and group-specific trajectories, with Bonferroni-adjusted pairwise comparisons used to identify significant differences between specific timepoints. Because quantitative demands were measured at only two time points (T0 and T1), a separate linear mixed-effects model was estimated for this outcome using a compound symmetry covariance structure to account for the correlation between the two repeated measurements within individuals. All statistical analyses were conducted using IBM SPSS Statistics v 29.01.

## 3. Results

Section 3.1 presents the overall changes in the perceptions of psychosocial work factors, work-related health and job satisfaction among professional service staff during the transition from full remote work to a hybrid work arrangement over 12 months. The results are presented chronologically in terms of the changes between baseline (T0, full remote work), 3-months (T1, employees returning to work in a hybrid work arrangement), and 12-months (T2, hybrid work arrangement). Section 3.2 presents the results for gender and household composition differences during the transition.

### 3.1 Changes in psychosocial work factors, work-related health and job satisfaction during the transition from full remote work to a hybrid work arrangement

Among the psychosocial work factors, there were no significant changes found in the perception of quantitative demands, social support from supervisor, and influence at work during the transition. However, significant overall changes were observed for social support from colleagues during the transition (F(2, 1093.32) = 3.06, p = .05). Bonferroni-adjusted pairwise comparisons indicated a significant increase between T1 (M = 78.61) and T2 (M = 81.78, p = .04), suggesting that social support from colleagues dipped during the initial transition to hybrid work before increasing and exceeding baseline levels at 12-months.

Significant changes were also observed for the work-related health factors during transition. Significant changes in exhaustion (F(2, 1052.03) = 5.07, p = .01) and stress (F(2, 1129.81) = 4.34, p = .01) were observed during the transition. Bonferroni-adjusted pairwise comparisons revealed a significant increase in exhaustion from T0 (M = 25.88) to T1 (M = 29.24, p = .005) followed by a partial but non-significant decrease at T2 (M = 27.55), suggesting that exhaustion increased during the initial transition and remained higher than baseline levels at 12-months. Bonferroni-adjusted pairwise comparisons for stress revealed a sustained increase during the transition, with pairwise comparisons indicating significant differences between T0 (M = 34.37) and T2 (M = 39.94, p = .02) but not between adjacent timepoints. There were no significant changes found in job satisfaction during the transition. Table 4 presents the results of the Type III tests of fixed effects for all outcomes whereas Table 5 presents estimated marginal means by timepoint and pairwise comparisons between timepoints for all outcomes, showing where significant change occurred across the transition period.

**Table 4:** Fixed effects from linear mixed-effects models for psychosocial work factors, work-related health outcomes and job satisfaction.

Outcome	Effect	F (df)	p
Quantitative demands	Time	1.69 (1, 584.53)ᵃ	.19
	Gender	4.63 (1, 753.33)	.03*
	Household composition	1.82 (3, 738.33)	.14
	Time × Gender	15.23 (1, 583)	<.001**
	Time × Household composition	.67 (3, 573.34)	.57
Social support from colleagues	Time	3.06 (2, 1093.32)	.05*
	Gender	.49 (1, 809.38)	.49
	Household composition	3.60 (3, 767.76)	.01*
	Time × Gender	2.87 (2, 1098.76)	.06
	Time × Household composition	.19 (6, 1065.57)	.98
Social support from supervisor	Time	1.42 (2, 1116.24)	.24
	Gender	18.04 (1, 829.79)	<.001**
	Household composition	5.58 (3, 786.26)	<.001**
	Time × Gender	3.54 (2, 1122.89)	.03*
	Time × Household composition	.49 (6, 1088.91)	.82
Influence at work	Time	2.07 (2, 1059.64)	.13
	Gender	30.65 (1, 801.35)	<.001**
	Household composition	2.14 (3, 765.88)	.09
	Time × Gender	3.71 (2, 1066.55)	.03*
	Time × Household composition	.95 (6, 1038.59)	.46
Exhaustion	Time	5.07 (2, 1052.03)	.01*
	Gender	5.98 (1, 779.62)	.02*
	Household composition	4.24 (3, 740.29)	.01*
	Time × Gender	2.14 (2, 1058.60)	.12
	Time × Household composition	.26 (6, 1026.10)	.95
Stress	Time	4.34 (2, 1129.81)	.01*
	Gender	9.17 (1, 795.02)	.003*
	Household composition	1.76 (3, 744.68)	.15
	Time × Gender	4.17 (2, 1135.49)	.02*
	Time × Household composition	.86 (6, 1095.16)	.53
Job Satisfaction	Time	.83 (2, 1106.07)	.44
	Gender	.004 (1, 797.37)	.95
	Household composition	3.36 (3, 755.81)	.02*
	Time × Gender	1.56 (2, 1112.57)	.21
	Time × Household composition	.31 (6, 1077.54)	.93

**Note:** All models estimated using restricted maximum likelihood (REML) with Satterthwaite-adjusted degrees of freedom. Outcomes measured at three timepoints used a first-order autoregressive [AR(1)] covariance structure. The estimated AR(1) autocorrelation parameters (ρ) ranged from.44 (stress) to.69 (influence at work) across outcomes, indicating moderate to strong within-person correlations across timepoints. ᵃ = Quantitative demands were measured at two timepoints, T0 (baseline, during full remote work) and T1 (3-months, during the transition to hybrid work). The compound symmetry covariance estimate for quantitative demands was 231.84. Bold values indicate statistically significant effects. p < .05 = *, p < .001 = **.

**Table 5:** Estimated marginal means by timepoint and pairwise comparisons between timepoints for psychosocial work factors, work-related health outcomes and job satisfaction.

Outcome	T0 M (se)	T1 M (se)	T2 M (se)	T0 - T1 ΔM (se), *p*	T0 - T2 ΔM (se), *p*	T1 - T2 ΔM (se), *p*
Quantitative demands ᵃ	39.57 (.92)	40.70 (1.03)	–	1.13 (.87)	–	–
Social support from colleagues	79.49 (1.04)	78.61 (1.19)	81.78 (1.29)	−.87 (1.12), p = 1.00	2.29 (1.43), p = .33	3.17 (1.28), p = .04*
Social support from supervisor	78.10 (1.25)	75.92 (1.43)	77.49 (1.56)	−2.18 (1.37), p = .33	−.61 (1.74), p = 1.00	1.57 (1.56), p = .95
Influence at work	50.59 (1.24)	51.31 (1.31)	52.85 (1.38)	.72 (.84), p = 1.00	2.26 (1.12), p = .13	1.54 (.97), p = .34
Exhaustion	25.88 (1.04)	29.24 (1.17)	27.55 (1.29)	3.36 (1.07), p = .01*	1.67 (1.40), p = .70	−1.69 (1.23), p = .52
Stress	34.37 (1.39)	38.36 (1.62)	39.94 (1.75)	3.99 (1.70), p = .06	5.57 (2.07), p = .02*	1.58 (1.93), p = 1.00
Job satisfaction	75.58 (.92)	74.78 (1.05)	73.95 (1.13)	−.80 (1.02), p = 1.00	−1.63 (1.27), p = .60	−.83 (1.15), p = 1.00

**Note:** T0 (baseline, during full remote work), T1 (3-months, during the transition to hybrid work) and T2 (12-months, hybrid work arrangement). ΔM = mean difference of later minus earlier timepoint, so positive values indicate an increase over time. Bold values indicate statistically significant effects. p < .05 = *, p < .001 = **. ᵃ = Quantitative demands were measured at two timepoints, T0 (baseline, during full remote work) and T1 (3-months, during the transition to hybrid work).

### 3.2 Gender and household composition differences in psychosocial work factors, work-related health and job satisfaction during the transition

Significant Time × Gender interactions during the transition were observed for quantitative demands, social support from supervisor, influence at work and stress (see Table 4), suggesting that men and women experienced different trajectories in these outcomes during the transition. The estimated marginal means for these significant gender differences are presented for each timepoint in Table 6.

**Table 6:** Estimated marginal means by gender at each timepoint for outcomes with significant time × gender interactions.

Outcome	T0 M (se)	T1 M (se)	T2 M (se)
**Quantitative demands**			
Women	39.74 (.94)	43.74 (1.02)	–
Men	39.40 (1.43)	37.66 (1.63)	–
**Social support from supervisor**			
Women	75.25 (1.28)	70.21 (1.40)	74.06 (1.47)
Men	80.96 (1.95)	81.64 (2.25)	80.93 (2.54)
**Influence at work**			
Women	47.97 (.93)	47.10 (1.00)	47.75 (1.05)
Men	53.21 (1.42)	55.51 (1.59)	57.94 (1.78)
**Stress**			
Women	34.99 (1.43)	43.14 (1.58)	43.57 (1.63)
Men	33.75 (2.17)	33.58 (2.56)	36.31 (2.86)

Women reported higher levels of quantitative demands than men at both timepoints (F(1, 753.33) = 4.63, p = .03). Although women and men reported similar levels of quantitative demands during full remote work at baseline (women, M = 39.74; men, M = 39.40) they showed significantly different trajectories when employees returned to work in a hybrid work arrangement at 3-months (F(1, 583) = 15.23, p < .001). During this period Bonferroni-adjusted pairwise comparisons revealed that women reported a significant increase in quantitative demands between full remote work at baseline (M = 39.74) and the transition to hybrid work at 3-months (M = 43.74, p < .001) while men reported a non-significant decrease in quantitative demands between full remote work at baseline (M = 39.40) and the transition to hybrid work at 3-months (M = 37.66, p = .21). Gender differences were also observed for social support from supervisors as women reported lower levels of support than men across all three timepoints (F(1, 829.79) = 18.04, p < .001) and showed significantly different trajectories during the transition (F(2, 1122.89) = 3.54, p = .03). Bonferroni-adjusted pairwise comparisons revealed that men reported stable levels of support during the transition with no significant changes between timepoints (T0, M = 80.96; T1, M = 81.64; T2, M = 80.93) whereas women experienced a significant decrease in support between full remote work at baseline (M = 75.25) and the transition to hybrid work at 3-months (M = 70.21, p < .001) before partially recovering in levels of support between the initial transition and during hybrid work at 12-months (M = 74.06, p = .02). Gender differences were also observed for influence at work as women reported lower levels of influence than men across all three timepoints (F(1, 801.35) = 30.65, p < .001) and showed significantly different trajectories during the transition (F(2, 1066.55) = 3.71, p = .03). Bonferroni-adjusted pairwise comparisons revealed that men reported increasing levels of perceived influence during the transition (T0, M = 53.21; T1, M = 55.51; T2, M = 57.94) however significant differences were only observed between full remote work at baseline and during hybrid work at 12-months (p = .03). However, women’s perceived influence remained unchanged during the transition with no significant differences observed (T0, M = 47.97; T1, M = 47.10; T2, M = 47.75).

In addition to this, gender differences were observed for stress as women reported higher levels of stress across all three timepoints (F(1, 795.02) = 9.17, p = .003) and showed significantly different trajectories during the transition (F(2, 1135.5) = 4.17, p = .02). Despite reporting similar levels of stress at baseline (women, T0, M = 34.99; men, T0, M = 33.75), women’s stress significantly increased between full remote work at baseline and the transition to hybrid work at 3-months (M = 43.14, p < .001) and remained significantly higher than baseline during hybrid work at 12-months (M = 43.57, p < .001). In contrast, men reported stable levels of stress throughout the transition with no significant differences observed between timepoints (T1, M = 33.58; T2, M = 36.31).

Significant differences in social support from colleagues, social support from supervisors, exhaustion and job satisfaction were observed between household composition groups (see Table 4). However, no significant time × household composition interactions were observed for any outcome, suggesting that all household groups followed similar trajectories over time. As these group differences were stable across timepoints, Table 7 presents the estimated marginal means averaged across time for the significant household composition main effects. Across the three timepoints, cohabitants with children reported the highest level of social support from colleagues (M = 82.98), social support from supervisors (M = 82.16), job satisfaction (M = 77.38) and the lowest level of exhaustion (M = 24.49) compared to the other household composition groups. Individuals living in single-person households reported the lowest level of social support from colleagues (M = 76.75), whereas individuals living in single-parent households with children reported the lowest level of social support from supervisors (M = 71.08), job satisfaction (M = 72.03), as well as the highest level of exhaustion (M = 30.85) across the three timepoints.

**Table 7:** Estimated marginal means by household composition for outcomes with significant main effects.

Outcome	Single-person household M (se)	Single-parent household with children M (se)	Cohabitant M (se)	Cohabitant with children M (se)
Social support from colleagues	76.75 (1.76)	79.68 (2.35)	80.42 (1.31)	82.98 (1.06)
Social support from supervisor	76.75 (2.10)	71.08 (2.82)	78.71 (1.57)	82.16 (1.27)
Exhaustion	30.03 (1.79)	30.85 (2.38)	24.85 (1.33)	24.49 (1.08)
Job satisfaction	73.26 (1.53)	72.03 (2.04)	76.42 (1.14)	77.38 (.92)

## 4. Discussions

This paper examined the perceptions of psychosocial work factors, work-related health, and job satisfaction among professional service staff during the transition from full remote work to a hybrid work arrangement over 12-months, along with the effects of gender and household composition. The result of the study suggests that there were a number of changes in professional service staff perceptions of the psychosocial work environment and job satisfaction during the transition to a hybrid work arrangement. The transition was associated with a significant deterioration in work-related health factors, as both exhaustion and stress increased during the transition. In addition to this, significant gender differences over time were observed for quantitative demands, social support from supervisors, influence at work, and stress, with women experiencing less favourable trajectories across all four outcomes. Moreover, overall differences in social support from colleagues, social support from supervisors, exhaustion, and job satisfaction were observed between household composition groups, however, these differences remained stable throughout the transition.

### 4.1 Overall perceptions of psychosocial work factors, work-related health and job satisfaction during the transition to a hybrid work arrangement

The results suggest that while overall changes in psychosocial work factors and job satisfaction were modest, a number of significant changes were observed during the transition to a hybrid work arrangement among professional service staff. There were no significant overall changes in the perceived level of quantitative demands, influence at work or social support from supervisors during the transition. Social support from colleagues showed a significant change over time, characterized by a slight dip during the initial transition to hybrid work followed by an increase that exceeded baseline levels at 12-months, however, the differences in the COPSOQ scores were minor. As professional isolation, particularly from one’s colleagues, has been identified as a common pitfall of telework, this finding suggests that the return to hybrid work may have provided opportunities to strengthen collegial relationships, supporting previous findings pointing to the resilience of professional services in managing the social challenges incurred by telework [30].

Professional service staff reported a significant deterioration in work-related health during the transition. Exhaustion increased significantly during the initial transition to hybrid work and, although it partially decreased at 12-months, did not return to baseline levels. Stress showed an even more concerning pattern, increasing progressively across the transition period and remaining elevated at 12-months. These findings suggest that the transition to hybrid work placed a sustained burden on employee well-being. Although studies have found an association between telework and negative work-related health outcomes [18], the association between telework and employee health is still far from clear [16]. No significant changes were observed in job satisfaction during the transition, suggesting that despite the increases in exhaustion and stress, employees’ overall evaluation of their work remained largely unaffected. Although the changes observed in the results of the study were small and fell below the threshold usually considered practically meaningful for COPSOQ scales [32], Pejtersen et al. [36] note that this threshold is conservative, and that even modest group-level changes can be practically meaningful. The consistent deterioration in work-related health during the transition, namely the rise in stress and the sustained elevation of exhaustion, can therefore be read as an early indicator calling for proactive monitoring and preventive institutional policy as hybrid work becomes embedded, particularly considering the gendered pattern observed.

### 4.2 The influence of gender and household composition on psychosocial work factors, work-related health and job satisfaction during the transition to a hybrid work arrangement

The results showed that there were significant gender differences over time during the transition to a hybrid work arrangement. Women reported increasing levels of quantitative demands while men reported a slight decrease, despite both groups starting at nearly identical levels during full remote work at baseline. Although quantitative demands were only measured twice, this divergence is notable as the baseline captured a period when full remote work was recommended, whereas the second timepoint at 3-months marked the beginning of the transition to a hybrid work arrangement. Women also reported significantly lower levels of social support from supervisors and influence at work compared to men, with these gaps widening during the transition. Women further reported a significant decline in social support from supervisors during the initial transition to hybrid work at 3-months before partially recovering during hybrid work at 12-months, while men’s perceptions remained stable throughout the transition. Furthermore, perceived influence at work among men increased steadily across the transition while it remained largely unchanged for women, resulting in a progressively widening gender gap. Our results, therefore, support previous studies highlighting that the transition to a hybrid work arrangement negatively impacted women’s perception of their psychosocial work environment more than among men [22,37,38].

The results also showed that women reported higher levels of exhaustion and stress during the transition compared to men, which is in line with previous studies showing that telework has a negative impact on women’s work-related health outcomes [31,39,40]. Despite starting at nearly identical levels during full remote work, stress among women increased sharply during the initial transition and remained elevated at 12-months, while stress remained largely stable throughout among men. Women also reported significantly higher levels of exhaustion across the transition, although the gender-specific trajectories over time did not differ significantly. Although the findings are in line with results from previous studies, it is important to note that the differences in men’s and women’s work-related health outcomes observed in this study may not necessarily be due to the transition to hybrid work, as pre-existing gender differences may have stayed stable during this time period. Although this study did not examine the structural relationship between psychosocial work factors and work-related health, a potential explanation for the negative impact of teleworking on women’s perception of their psychosocial work environment and work-related health could be due to the flexibility paradox. The flexibility paradox describes the paradoxical effects of the high levels of autonomy and control found in telework and argues that it leads to work intensification through the blurring of the boundaries between the personal and professional life, which affects women more than men due to traditionalized divisions of labour in the household [41,42]. Furthermore, studies have shown that the relationship between telework and work-related health outcomes among women is mediated by work-family conflicts and the inability to psychologically disengage from work [43]. It is, however, important to acknowledge that the negative impact of the flexibility paradox may be lessened in gender equal societies such as Sweden, which may explain why the observed gender differences in our results are relatively small [44]. Lastly, our results showed no significant gender differences in job satisfaction during the transition.

Our findings also point to the role of household composition in shaping perceptions of psychosocial work factors, work-related health and job satisfaction during the transition to hybrid work. Significant overall differences between household groups were observed for social support from colleagues, social support from supervisors, exhaustion and job satisfaction. However, as no significant time × household composition interactions were found for any outcome, these differences reflect stable group-level patterns rather than different trajectories during the transition itself. During the overall transition period, cohabitants with children consistently reported the highest levels of social support from colleagues and supervisors, the highest levels of job satisfaction and the lowest level of exhaustion. In contrast, those living in single-person households and single-parent households with children reported less favourable levels of these outcomes. Single-parent households reported the lowest levels of social support from supervisors and job satisfaction and the highest levels of exhaustion, while single-person households reported the lowest levels of social support from colleagues. These household composition differences could potentially be explained through the lens of social isolation and the flexibility paradox. The less favourable perceptions of psychosocial work factors, which mostly pertain to social interactions, held by those living in single-person households reporting could be indicative of their feelings of social isolation induced by telework [15]. Although the use of ICT can facilitate communication during telework, the perceived frequency of communication can both enhance and alleviate perceptions of isolation [45]. Studies have also shown that low levels of perceived social support from supervisors can also increase social isolation among employees via reduced knowledge sharing [14]. Although the study did not statistically model structural relations, these findings could potentially explain the less favourable work-related health outcomes and lower levels of job satisfaction, a sequence typically proposed by work-stress models such as the Job Demands-Resource model [46]. The less favourable perceptions of psychosocial work factors held by those living in single-parent households with children tentatively suggest that the flexibility afforded in a hybrid work arrangement yields differential effects depending on household composition. Studies have shown that the nature of one’s household composition in terms of the age of dependents can impact work impairment during telework [47] and that having dependent children can facilitate both positive and negative employee outcomes [48].

### 4.3 Limitations

This study has several limitations. Firstly, the study measures two psychosocial work factors (social support from supervisor and social support from colleagues) using single items. However, the use of the mandatory core item in each scale, used in the present study, is required when using shortened versions of COPSOQ scales [33]. Moreover, the results show slight changes in the scores on the COPSOQ scales, and there needs to be a difference of at least 5–10 points in the scales for the difference to be meaningful [32]. Moreover, this study uses a sample from one medical faculty in Sweden, which limits the generalizability of the findings. However, by doing so, the study does address key gaps in the literature by exploring how perceptions of psychosocial work factors, work-related health, and job satisfaction changes during the transition from full remote work to a hybrid work arrangement over time, using an understudied population – professional service staff. As our analysis did not explore structural relationships, the findings in the study can only highlight the observed differences without making inferences as to why they occur. Lastly, our results highlight the transition from full remote work to a hybrid work arrangement, and therefore, the results do not apply to transitions from office work to hybrid work.

### 4.4 Conclusions and practical implications

Our results showed that while overall changes in psychosocial work factors and job satisfaction were modest, a number of significant changes were observed during the transition to a hybrid work arrangement among professional service staff. The results highlighted negative work-related impacts during the initial transition period which then evened out after employees got used to working in a hybrid work arrangement. This suggests that it is important to give hybrid work practices enough time to settle before examining their work-related effects. The results also showed that gender and household composition may be important factors that influence the work-related associations of hybrid work. This highlights how managers and organizations need to find strategies to address the gendered patterns found in the work-related effects of hybrid work to prevent it from diminishing the gender equality in workplaces [49]. Researchers such as Arabadjieva and Franklin [50] have called for additional legislative support to help organizations become more gender-sensitive and equitable in the implementation of hybrid work arrangements. The study’s findings, along with previous studies (e.g., 31), suggest that higher education institutions should implement action plans for gender equality in hybrid work arrangements. Furthermore, our results highlight the need for management training in virtual leadership and family-supportive behaviours to ensure that the benefits of hybrid work arrangements are accessible to all employees [51,52].
